# Supplementary material for: Plant Growth Promotion and Stress Tolerance Enhancement through Inoculation with Bacillus proteolyticus OSUB18
Source: Biology (Basel). 2023 Dec 6;12(12):1495. doi: 10.3390/biology12121495 (PMC10740446; doi:10.3390/biology12121495)
Supplement: Supplementary file 1 [file biology-12-01495-s001.zip › Supplemental Table S1.pdf]

Table S1 Phytopathogenic microbes used in this study.

Microbe	Microbial Species	Plant Disease	Reference
Bacteria	*Pseudomonas syringae*	Bacterial speck	[32]
Fungi	*Botrytis cinerea*	Gray mold disease	[4]
Fungi	*Fusarium graminearum*	Fusarium head blight disease	[43]
Fungi	*Fusarium oxysporum*	Fusarium wilt disease	[44]
Fungi	*Magnaporthe oryzae*	Rice blast disease	[45]
Oomycete	*Phytophthora capsici*	Blight and fruit rot disease	[46]
Oomycete	*Pythium ultimum*	Damping off and root rot disease	[46]
