# Supplementary material for: Plant Growth Promotion and Stress Tolerance Enhancement through Inoculation with Bacillus proteolyticus OSUB18
Source: Biology (Basel). 2023 Dec 6;12(12):1495. doi: 10.3390/biology12121495 (PMC10740446; doi:10.3390/biology12121495)
Supplement: Supplementary file 1 [file biology-12-01495-s001.zip › Supplementary caption.pdf]

Table S1 Phytopathogenic microbes used in this study.

Table S2 Transcript levels of genes induced (log2 fold change > 1, p-value adjusted < 0.05) in Arabidopsis plants inoculated with OSUB18 compared to water control. An Excel file was submitted separately. The Log2 fold change values were labeled with gradient colors in the excel sheet.

Table S3 Transcript levels of genes repressed (log2 fold change > 1, p-value adjusted < 0.05) in Arabidopsis plants inoculated with OSUB18 compared to water control. An Excel file was submitted separately. The Log2 fold change values were labeled with gradient colors in the excel sheet.
